# Visceral fat and attribute-based medicine in chronic kidney disease

**DOI:** 10.3389/fendo.2023.1097596

**Published:** 2023-02-09

**Authors:** Hiroshi Kataoka, Kosaku Nitta, Junichi Hoshino

**Affiliations:** Department of Nephrology, Tokyo Women’s Medical University, Tokyo, Japan

**Keywords:** visceral fat, patient-centered medicine, sex difference, personalized medicine, obesity, precision medicine, chronic kidney disease, attribute-based medicine

## Abstract

Visceral adipose tissue plays a central role in obesity and metabolic syndrome and is an independent risk factor for both cardiovascular and metabolic disorders. Increased visceral adipose tissue promotes adipokine dysregulation and insulin resistance, leading to several health issues, including systemic inflammation, oxidative stress, and activation of the renin-angiotensin-aldosterone system. Moreover, an increase in adipose tissue directly and indirectly affects the kidneys by increasing renal sodium reabsorption, causing glomerular hyperfiltration and hypertrophy, which leads to increased proteinuria and kidney fibrosis/dysfunction. Although the interest in the adverse effects of obesity on renal diseases has grown exponentially in recent years, the relationship between obesity and renal prognosis remains controversial. This may be attributed to the long clinical course of obesity, numerous obesity-related metabolic complications, and patients’ attributes. Multiple individual attributes influencing the pathophysiology of fat accumulation make it difficult to understand obesity. In such cases, it may be effective to elucidate the pathophysiology by conducting research tailored to individual attributes from the perspective of attribute-based medicine/personalized medicine. We consider the appropriate use of clinical indicators necessary, according to attributes such as chronic kidney disease stage, level of visceral adipose tissue accumulation, age, and sex. Selecting treatments and clinical indicators based on individual attributes will allow for advancements in the clinical management of patients with obesity and chronic kidney disease. In the clinical setting of obesity-related nephropathy, it is first necessary to accumulate attribute-based studies resulting from the accurate evaluation of visceral fat accumulation to establish evidence for promoting personalized medicine.

## 1 Introduction

Accumulated epidemiologic evidence indicates that being overweight and obese are risk factors for chronic kidney disease (CKD) (1–4) and end-stage kidney disease (ESKD) (5–8); additionally, the causal link between obesity and CKD has been extensively reviewed (9–11). Visceral fat accumulation is the central pathological condition in obesity/metabolic syndrome (12–15) and is significantly associated with atherosclerosis (14), hypertension (16, 17), and metabolic impairments, including hyperglycemia/diabetes mellitus (17–19), hypertriglyceridemia (17), low high-density lipoprotein (HDL) cholesterol (17, 20), hyperuricemia (21, 22), high C-reactive protein concentration (14, 17), fatty liver (14), cardiovascular disease (CVD) (23), and kidney disease (24, 25). Nevertheless, at present, no clinical practice guidelines for obesity-related glomerulopathy (ORG) have been established. This narrative review provides an overview of visceral fat and obesity-related kidney disease and its clinical indicators, aiming to generate novel ideas for future studies and clinical applications focusing on attribute-based medicine/personalized medicine.

## 2 Literature review

### 2.1 Visceral fat is a major pathophysiological condition of obesity/metabolic syndrome

The pathophysiology of obesity/visceral fat accumulation is complex, with numerous interrelated aspects, including a sedentary lifestyle, individual dietary habits, genetic predisposition, and environmental factors (26–29). Visceral adipose tissue (VAT) plays a central role in being overweight and obese (30–34), whereas subcutaneous fat tissue is considered benign or protective (35, 36). Increased visceral fat accumulation causes adipose tissue inflammation and adipokine dysregulation (30–34), which can lead to dyslipidemia, insulin resistance (32, 37), chronic systemic inflammation (32, 38, 39), oxidative stress (30), brain melanocortin system stimulation (38, 40), sympathetic nervous system overactivation (40–42), renin-angiotensin-aldosterone system (RAAS) overactivation (43–47), mineralocorticoid receptor activation (48), sodium retention (49, 50), and extracellular fluid volume expansion (50–52). Increased visceral fat accumulation is also accompanied with perirenal and renal sinus fat accumulation, which causes high intrarenal pressure, which leads to compression of the vasa recta capillaries and thin loops of Henle, reduced blood flow in the renal medulla, increased sodium reabsorption in the loop of Henle, RAAS activation, and increased sodium reabsorption (50, 53, 54). These pathological conditions interact in a complex manner, ultimately damaging the kidneys by causing glomerular hyperfiltration (55, 56) and inflammation (57, 58), both of which are characteristics of obesity-related kidney disease (50, 53, 54, 59–61).

### 2.2 The complex pathophysiology of obesity

Numerous studies in the last 20 years have investigated obesity, significantly elucidating the systemic pathology associated with visceral adiposity/obesity and the mechanism of kidney injury in patients with obesity (2, 62). However, while the number of patients with obesity and patients with ORG has continued to increase, treatment strategies for ORG generally have remained ineffective in clinical practice (63, 64). Although patients and medical staff understand that weight loss is a simple solution to obesity-related diseases, the clinical prognostic indicators for ORG are poorly established, as is the optimal treatment for individual patients, with no clinical practice guidelines for ORG (63, 65, 66).

The following issues may have led to some confusion in studies and the creation of a knowledge gap regarding obesity-related neuropathy (1): the concept of the “obesity paradox,” in which protective effects of obesity have been observed in certain patient populations [e.g., ESKD patients (67, 68)] (2); the idea of a “metabolically healthy obesity phenotype” (69, 70) (3); the biphasic clinical change in the estimated glomerular filtration rate (eGFR) based on hyperfiltration during CKD progression in patients with obesity (56, 71) (4); the biphasic course of glomerular size during glomerular damage (72, 73) (5); the presence of many obesity-related complications and the long clinical course of obesity; and (6) the lack of evidence based on the precise measurement of visceral fat. Among these, though the concept of the “obesity paradox” remains controversial (67, 74–76), its existence has recently been questioned owing to concerns about the limitation of epidemiological studies (i.e., selection biases, confounding factors, influence of malnutrition), the inherent limitations of anthropometric measures, such as the body mass index (BMI), and the limitations of studies with short periods of observation (74, 77–82). As Kramer et al. (83) reported, multiple residual confounders and biases strongly affect the “obesity paradox.” Indeed, bariatric surgery, effective for multiple residual confounders, such as obesity-related complications, solves the “obesity paradox” by decreasing visceral fat accumulation and glomerular hyperfiltration, which are essential pathophysiological conditions of obesity (84, 85). Furthermore, the concept of a “metabolically healthy obesity phenotype” is also questioned by an accumulation of results considering the long clinical course of obesity. The “metabolically healthy obesity phenotype” is reportedly associated with low levels of VAT/ectopic fat, high levels of lower body subcutaneous fat storage, younger age, insulin sensitivity, increased adiponectin, a favorable lipoprotein profile, and non-Hispanic black race/ethnicity (18, 86). Meta-analyses of studies with a follow-up duration >10 years reported that individuals with “metabolically healthy obesity phenotype” are at an increased risk for CVD events (87, 88), with this risk increasing with a longer follow-up duration (89). The accumulation of studies that precisely evaluates visceral fat is an issue that remains to be addressed by researchers and clinicians worldwide.

### 2.3 Precise visceral fat measurement in patients with CKD

Although there are various anthropometric and imaging measurement methods clinically available to assess adiposity (51, 80, 90), to fully clarify the pathophysiological condition in obesity, it is important to determine whether volumetric fat measurements can accurately characterize the heterogeneity of abdominal fat distribution between individuals (91). BMI and waist circumference are easy to examine and have been widely used to define obesity and abdominal obesity. Waist circumference has been used as an indicator closely associated with visceral fat (92). However, theoretically, both BMI and waist circumference cannot be used to distinguish between visceral and subcutaneous fat mass. Since VAT and SAT differ greatly in their functional significance and response to weight gain, anthropometric data alone is not sufficient for an accurate risk assessment of adiposity (80). Therefore, imaging methods need to be developed to identify individuals with excessive visceral adiposity (51). Imaging measurement of adiposity can be performed by various methods, including computed tomography (CT), magnetic resonance imaging (MRI), dual-energy X-ray absorptiometry, and electrical bioimpedance (93). Among these, only CT accurately measures visceral fat area (VFA) (94). CT can be performed rapidly and interpreted to segment adipose tissue deposits and measure their area or volume (80). CT produces high-resolution images conveniently and with high repeatability (94), providing accurate localization data (95). Although CT is expensive and exposes the patient to radiation, quantitative CT is currently the technology of choice for the measurement and analysis of VFA (96). At present, though CT and MRI are ideal methods to assess adiposity in clinical research (51, 93, 97), studies using visceral fat assessment evaluated by these techniques are still lacking.

### 2.4 Attribute-based medicine for personalized medicine

In recent years, the concept of personalized medicine/precision medicine/tailored medicine has been developed alongside the concepts of patient-centered medicine (98, 99). In a clinical setting, personalized medicine can provide access to knowledge that either validates or alters a medical decision from one that is based on the evidence for the average patient to one that is based on the individual’s unique characteristics/attributes (100). In personalized medicine, patients are treated individually according to their individual heterogeneous characteristics (101, 102), with the advantages of the disaggregation of data and analyses of differences within sub-cohorts having been reported (102, 103). Recently developed artificial intelligence/machine learning has the potential to bring about the ideal personalized medicine (100, 104, 105). However, there are still issues that need to be resolved to establish personalized medicine for patients with CKD (100, 104). Fröhlich et al. identified the following challenges in data science (artificial intelligence [AI]/machine learning) for personalized medicine (1): insufficient prediction performance for clinical practice (2), difficulties in interpretation, and (3) insufficient validation for clinical practice (105). Indeed, unlike in genetic diseases (106) where personalized medicine can be applied according only to genetic mutations (107–110), most patients with CKD are affected by multiple risk factors for disease progression (111, 112). In patients with CKD, the risk factors and pathophysiological conditions generally differ regarding patient attributes (113). In patients with obesity and CKD, multiple attributes, including age (114, 115), sex (116–119), race/ethnicity (17, 120), fat distribution (119, 121), the amount of VAT (119, 121, 122), and CKD stage (115), are known to more intricately influence the pathophysiology of fat accumulation and cardiorenal metabolic disease (51, 80). Such complex interactions of chronic diseases with obesity raise the difficulty of interpretation of pathophysiology, prognosis prediction, and validation in a clinical setting.

In such a multifactorial disease like CKD, we consider that attribute-based medicine (113, 123), supported by attributes/characteristics (100, 105) such as sex and age, is useful for the establishment of personalized medicine. That is, instead of jumping from traditional medicine in an entire cohort to personalized medicine in individuals, we interpose a step (attribute-based medicine) to bridge both approaches. Attribute-based medicine can help solve the challenges enumerated by Fröhlich et al. (105) by increasing the future accuracy of machine learning predictions, enabling patients and clinicians to interpret machine learning-generated predictions, and making it easier to validate in the clinical setting. Attribute-based medicine may provide a bridge between traditional statistical research and personalized medicine.

### 2.5 Attribute-based medicine for patients with obesity and CKD

Before devising personalized medicine approaches in patients with CKD, high-quality databases must be created and risk factors for the acceleration of the CKD progression must be identified (113, 115, 118, 119, 124–129), paying attention to attributes such as sex differences or ages (130, 131). Indeed, it has been reported that even data used in AI should be divided according to sex and attribute, which makes collecting data disaggregated by age and sex essential if AI is to fulfill its promise of improving outcomes for everyone (132–135). Therefore, from the standpoint of patient-centered medicine, women and the elderly should be treated based on research evidence from female (131, 136–139) and geriatric cohorts (140–142), respectively. In chronic diseases, sex and age are important modifiers of pathophysiology and disease development. However, data disaggregated by age, sex, or obesity are still scarcely available from prospective studies (132, 137). To establish research supporting precision/personalized medicine, it is necessary to conduct further large-scale studies which include the analyses of disaggregated data (143).

In cardiology, sex-specific CVD risk assessment using CT or MRI-based fat measures has already been validated (144, 145). In nephrology, human studies on visceral fat and kidney prognosis have established clear evidence for kidney prognosis, especially regarding sex-specific differences (51, 144, 146). Several indicators that reflect obesity, such as BMI, waist circumference, VFA, and the visceral-to-subcutaneous fat ratio (V/S ratio), seem to explain CKD progression. However, the rationale and merits of various indicators likely vary and are insufficient to establish strong evidence (119, 147–152). Therefore, to address the gaps in knowledge regarding the pathophysiology of obesity and its impact on kidney disease, it will be important to accurately assess volumetric fat measurements to clearly characterize the heterogeneity of abdominal fat distribution between individuals and the differences in fat distribution between sexes (91). In this regard, CT- or MRI-based measures should be more indicated to study the effect of VAT on kidney disease.

## 4 Discussion

### 4.1 Attribute-based medicine for patients with CKD and obesity: A consideration of the sex differences in visceral adiposity and CKD progression

Among the multiple attributes influencing the pathophysiology of fat accumulation, sex differences in visceral adiposity and CKD progression are particularly important. Firstly, sex hormones have important roles in the accumulation and distribution of body fat (153). As a result, fat distribution significantly differs between the sexes, as men have relatively more visceral fat and women have relatively more subcutaneous fat (36, 154). Furthermore, men have higher levels of visceral fat (155) than premenopausal women, with the decline in estrogen levels upon menopause being associated with an increase in visceral fat in women (156). Post-menopause, the amount of estrogen secreted from the ovaries dramatically diminishes, resulting in a decrease in brain anorexigenic signaling through estrogen, evoking the storage of lipids in visceral fat, a major source of estrogen in postmenopausal women (157, 158).

Secondly, premenopausal women are generally protected from CVDs due to the activation of RAAS, with a previously established involvement of estrogen in this mechanism (159, 160). Although the angiotensin-converting enzyme/angiotensin II/angiotensin receptor 1 (ACE/Ang II/AT1R) axis plays a major role in the classic renin-angiotensin signaling pathway, namely in water and salt retention, vasoconstriction, and in proliferative, proinflammatory, and profibrotic processes (161), estrogen has been reported to reduce the activation of that axis (162, 163). Estrogen reduces ACE activity (164), AT1R expression (165, 166), and aldosterone production in animal models (167). Men and postmenopausal women have higher renin activity and levels (168, 169), as well as increased plasma aldosterone levels (170), than premenopausal women. These increases in RAAS activation and visceral fat in postmenopausal women can be avoided by estrogen replacement therapy (169, 171–173). Furthermore, estrogen shifts the balance toward the AT2R/ACE2/Ang- (1–7)/mitochondrial assembly receptor (MasR) axis [the protective/depressor renin-angiotensin signaling pathways (174)], which opposes the pressor actions of AT1R (160). Obesity is associated with the activation of the ACE/Ang II/AT1R axis (175, 176), with the overactivation of Ang II in obesity stimulating AT1R to promote hypertension, insulin resistance, and energy imbalance (176). However, the protective estrogen-RAAS interactions *via* AT2R/ACE2/Ang- (1–7)/MasR appear to be diminished by obesity (177) and aging (177), suggesting that the protective effect against CVD in women may be attenuated by an increase in visceral fat. Indeed, in human studies, the various vasoprotective effects of estrogen, including vasodilation, anti-inflammatory properties, and lipid profile decline, are nonexistent in hyperglycemic states and obesity (178–180). Features associated with obesity or metabolic syndrome in women generally emerge after menopause (181, 182), which may induce a concurrent progression of CKD (183–186).

Men with CKD generally have a worse prognosis than women, which leads to a substantially higher proportion of men with ESKD (187–189). Women seem to be protected against the development and progression of CKD (183, 190, 191), and the presence of estrogen further protects against kidney injury (192). Although the pathological mechanism underlying the sex-specific differences in CKD has not yet been completely elucidated, sex-specific differences in visceral fat accumulation (157, 158) are associated with sex-specific differences in CKD progression (20, 115, 119, 193, 194). For example, in a representative multicenter CKD study in Japan, using a ≥50% eGFR decline or ESKD as the endpoints, the sex-based Kaplan–Meier survival curves revealed that the kidney survival rate was significantly lower in men than in women among nonelderly patients (age <65 years) (113).

### 4.2 Attribute-based medicine for patients with CKD and obesity: A consideration of the cutoff of VFA 100 cm^2^ in visceral adiposity

In Japan, the clustered number of metabolic syndrome components is greater than 1.0 for individuals with a VFA ≥100 cm^2^ (13), with the best combination of sensitivity and specificity for determining patients with multiple risk factors identified for a VFA cutoff of 100 cm^2^ (13). Furthermore, VFA ≥100 cm^2^ is used as a diagnostic criterion for metabolic syndrome in Japan (12), with patients having VFA ≥100 cm^2^ being at risk for cardiovascular (195, 196), coronary artery (197), and cerebral small vessel (198) diseases. Although, generally, there are sex differences in waist circumference criteria for metabolic syndrome (199), it has been reported that there is no sex difference in the metabolic significance of the amount of visceral fat (196, 200). The mean number of obesity-related cardiovascular risk factors exceeded 1.0 at 100 cm^2^ of VFA both in men and women (196). These results indicate the significance of differentiating patients according to a 100 cm^2^ VFA threshold (201), regardless of sex, as well as highlight the need for studies based on the 100 cm^2^ threshold VFA value.

In kidney disease, the presence of metabolic syndrome (202) and a VFA ≥100 cm^2^ (115) are associated with CKD progression. Interestingly, a VFA ≥100 cm^2^ significantly interacted with the V/S ratio in terms of the renal prognosis (119). As metabolic complications are increased with a VFA ≥100 cm^2^ (115), the significance of VFA or V/S ratio in patients with a VFA ≥100 cm^2^ seems to become relatively less important. However, considering that many metabolic complications develop based on obesity, patients with a VFA ≥100 cm^2^ need not only medical intervention for each metabolic disease but also a reduction of excessive visceral fat itself (200).

### 4.3 Attribute-based medicine for patients with CKD and obesity: A consideration of the aging in visceral adiposity and CKD progression

As menopause influences obesity among women, it is clinically important to consider the influence of aging itself. Indeed, for women aged <55 years, which includes both pre- and menopausal statuses, VFA is markedly lower (median value, 59.8 cm^2^), with considerably fewer obesity-related cardiovascular risk factors, than for women ≥55 years of age or men (196). The average VFA increased with age in both men and women, above the 100 cm^2^ threshold after the age of 40 years in men, and close to the 100 cm^2^ threshold after the age of 60 years in women (196), with the mean number of obesity-related cardiovascular risk factors being >1.0 at ages 40 years in men and 60 years in women (196). Therefore, prevention of obesity-related diseases is required at an earlier stage for men than for women (200). On the other hand, though the incidence of CVDs in women lags behind men by 10 to 20 years (203), women generally live longer than men (160). Therefore, obesity management in postmenopausal women should not also be neglected.

Systemic renin and aldosterone levels decrease with age due to decreased renin production and release (204). It has been reported that older individuals have lower plasma renin and aldosterone levels compared with younger controls (205, 206), with impaired responses to RAAS stimuli, such as sodium depletion, hyperkalemia, and upright posture (207, 208), in older individuals (especially in late-elderly individuals). Generally, the rate of CKD progression is slow in elderly individuals (209–212). Although the reason for this has not been elucidated in human clinical studies, we consider that decreased systemic RAAS activation/glomerular hyperfiltration/glomerular hypertrophy axis (73, 129, 204) may be a factor. Although, the presence of diabetes mellitus (DM) (209, 213) and an increased BMI (213) are associated with kidney disease progression in elderly individuals, when patients with CKD are analyzed using cross-classification approach in detail (113), interestingly, DM alone was not an aggravating factor for renal prognosis in non-obese patients with CKD aged ≥65 years. In patients with CKD aged ≥65 years, poor kidney prognosis was observed only when both DM and obesity were present (113). This implies a decrease in RAAS activation in patients with CKD aged ≥65 years, and simultaneouslysuggests that attention should be paid to the overlapping of obesity and DM even in the elderly. The age-based Kaplan–Meier survival curves revealed that the kidney survival rate was significantly lower in obese patients with DM and a BMI ≥25 kg/m^2^ (4 years survival, 57.8%) than in non-obese patients with DM and a BMI <25 kg/m^2^ (4 years survival, 70.7%) (113). As RAAS overactivation (43–47) is one of the important pathologies contributing to obesity/metabolic syndrome and DM, the effects of RAAS activation among elderly individuals with obesity should be examined more specifically in future studies.

### 4.4 Challenges for attribute-based medicine for obesity-related kidney disease

Currently, the biggest challenge in promoting attribute-based medicine for patients with CKD is the lack of evidence regarding visceral fat and kidney disease progression. We conducted a literature search in the PubMed database in December 2022 using the keywords “visceral fat,” “kidney,” and “outcome,” which yielded 130 relevant articles. Among these, only three studies from two cohorts reported statistically significant associations between obesity evaluated by visceral fat measured using CT or MRI and CKD progression (kidney function decline) over a >2-year longitudinal observation period (**Table 1**). One of these studies, from the cohort reported by Madero et al. (148), confirmed the association between VFA measured on CT and kidney function decline, defined as a decrease in eGFR of >30% during a median follow-up of 8.9 years; their recruited patients were limited to individuals aged 70–79 years (**Table 1**, upper line). The other two reports were from our cohort (115, 119). Manabe et al. (115) found that VFA was significantly associated with CKD progression in a cohort with a wide age range (mean age, 59.2 years). The hazard ratios of VFA regarding CKD progression were higher in patients with VFA <100 cm^2^ than in patients with VFA ≥100 cm^2^ but did not differ between sexes (**Table 1**, middle line). The study by Kataoka et al. (119) was the first to show that the V/S ratio was significantly associated with CKD progression, particularly in the sub-cohort of VFA <100 cm^2^ compared with that of VFA ≥100 cm^2^ (P-value for interaction <0.01). Additionally, the hazard ratios of the V/S ratio regarding CKD progression were higher in women than in men (**Table 1**, lower line). Therefore, in women and patients with low visceral adiposity, the V/S ratio appears to be an early indicator of CKD progression. In this manner, the studies on visceral fat measured by CT are suggestive of an association between visceral fat accumulation and CKD progression. However, sufficient evidence is not present to guide clinical decision-making; further studies with longer observation periods are necessary to detect unhealthy obesity. Furthermore, as patients with obesity or advanced CKD generally have many complications and risk factors (113), we expect that large-scale studies that appropriately manage confounding factors will be reported in the future.

**Table 1:** Risks of visceral fat indicators for CKD progression.

Study	Variables	Patients	Study endpoint	Follow-up	OR/HR (CI 95%) in the entire cohort and sub-cohorts
Madero et al. (148)	VFA (the highest quartile)	2489 individuals aged 70–79 years	a ≥ 30% eGFR_cysC_ decline	8.9 years	1.4 (1.0–1.9)
Manabe et al. (115)	VFA (10 cm^2^ increase)	200 patients with CKD	a ≥ 50% eGFR_cre_ decline or ESKD	12.3 years	1.1 (1.0–1.1) 1.1 (1.0–1.1) in women 1.1 (1.0–1.1) in men 1.3 (1.0–1.8) in VFA < 100 cm^2^ 1.1 (1.0–1.1) in VFA ≥ 100 cm^2^
Kataoka et al. (119)	V/S ratio	200 patients with CKD	a ≥ 30% eGFR_cre_ decline or ESKD	12.8 years	1.8 (1.0–2.9) 2.4 (1.0–4.6) in women 1.1 (0.6–2.1) in men 6.4 (2.4–17.3) in VFA < 100 cm^2^ 1.0 (0.5–2.0) in VFA ≥ 100 cm^2^

CKD, chronic kidney disease; Follow-up, median follow-up duration; OR, odds ratio; HR, hazard ratio; CI, confidence interval; VFA, visceral fat area; eGFR_cysC_, estimated glomerular filtration rate based on cystatin C; eGFR_cre_, estimated glomerular filtration rate based on creatinine; ESKD, end-stage kidney disease requiring dialysis; V/S ratio, visceral-to-subcutaneous fat ratio evaluated using computed tomography.

## 5 Perspective

Attribute-based medical care and research are the first steps to developing personalized medicine. However, at present, attribute-based medical care is not widespread enough to provide individual medical care in a clinical setting. Although much has been elucidated about the pathophysiology of kidney injury in patients with obesity, data from human studies on visceral fat and kidney prognosis are insufficient to establish the necessary evidence for attribute-based medicine in obesity-related renal pathologies. The accumulation of larger and longer-term studies focusing on specific attributes is necessary to resolve the existing controversy, especially concerning sex-specific kidney disease prognosis.

## Author contributions

HK performed the literature search and wrote the manuscript. KN and JH were involved in planning and supervising the work. All authors contributed to the article and approved the submitted version.
